# PERSEUS 24-month analysis: a prospective non-interventional study to assess the effectiveness of intravitreal aflibercept in routine clinical practice in Germany in patients with neovascular age-related macular degeneration

**DOI:** 10.1007/s00417-021-05073-8

**Published:** 2021-02-06

**Authors:** Nicole Eter, Zoran Hasanbasic, Georgios Keramas, Christine Rech, Helmut Sachs, Harald Schilling, Joachim Wachtlin, Peter Wiedemann, Carsten Framme, for the PERSEUS Study Group

**Affiliations:** 1 Klinik für Augenheilkunde, Universitätsklinikum-Augenklinik Münster, Domagkstr. 15, 48149 Münster, Germany; 2 Bayer Vital GmbH, Leverkusen, Germany; 3 Data Generation, Bayer Vital GmbH, Leverkusen, Germany; 4 Augenklinik, Städtisches Klinikum Dresden-Friedrichstadt, Dresden, Germany; 5 Klinik für Augenheilkunde, St.-Johannes-Hospital, Dortmund, Germany; 6 Abteilung für Augenheilkunde, Sankt Gertrauden-Krankenhaus, Berlin, Germany; 7 MHB, Medizinische Hochschule Brandenburg, Neuruppin, Germany; 8 Klinik und Poliklinik für Augenheilkunde, Universitätsklinikum Leipzig, Leipzig, Germany; 9 Klinik für Augenheilkunde, Medizinische Hochschule Hannover, Hannover, Germany

**Keywords:** Age-related macular degeneration, nAMD, wAMD, Neovascular, VEGF, Intravitreal, Aflibercept, Real-world evidence

## Abstract

**Purpose:**

To evaluate the real-world effectiveness of intravitreal aflibercept injections in Germany in patients with neovascular age-related macular degeneration over 24 months.

**Methods:**

PERSEUS was a prospective, non-interventional cohort study. The primary endpoint was the mean change in visual acuity (VA) from baseline. Secondary endpoints included the proportion of patients with a VA gain or loss of ≥ 15 letters and the frequency of injections and examinations. Patients with regular (bimonthly after 3 monthly injections during year 1 and ≥ 4 injections in year 2) and irregular (any other) treatment were analyzed. The last observation carried forward (LOCF) and the observed cases (OC) approach was applied for primary endpoint analysis to account for missing data.

**Results:**

803 patients were considered for effectivity analysis. At month 24, only 38% of the patients were still under observation. The LOCF population included 727, the OC population 279 patients. Treatment-naïve patients improved by 6.3 (LOCF)/8.1 (OC) letters with regular treatment over 24 months but only by 3.3 (LOCF)/3.1 (OC) letters with irregular treatment. The proportion of treatment-naïve patients achieving a VA improvement of ≥ 15 letters was similar between regularly and irregularly treated cohorts. However, considerably more patients in the irregular cohorts experienced a VA worsening of ≥ 15 letters than in the regular cohorts (LOCF: 18.7% vs. 7.4%).

**Conclusions:**

Regular IVT-AFL treatment resulted in better VA outcomes than irregular treatment at month 24. However, only a minority of patients received regular treatment over a 2-year period.

**Supplementary Information:**

The online version contains supplementary material available at 10.1007/s00417-021-05073-8.

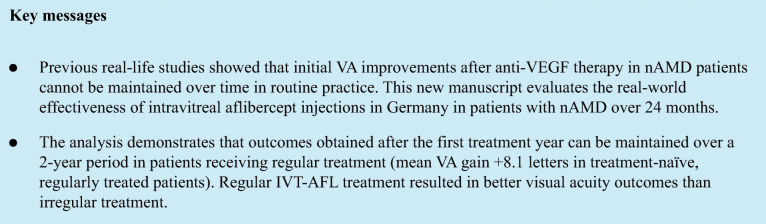

## Introduction

Intravitreal anti-vascular endothelial growth factor (anti-VEGF) therapy is the standard of care for treating neovascular age-related macular degeneration (nAMD). Randomized controlled trials with the approved anti-VEGF agents ranibizumab (MARINA [1], ANCHOR [2]) and aflibercept (VIEW1 and VIEW 2) [3] showed an improvement both in visual acuity (VA) and in morphological parameters. However, real-life studies with ranibizumab showed that initial VA improvements cannot be maintained over time in routine practice and outcomes were less favorable than in the pivotal controlled trials (AURA [4, 5], WAVE [6], COMPASS [7]).

The PERSEUS study (Prospective Non-intERventional Study to Assess the Effectiveness of Aflibercept in roUtine Clinical Practice in PatientS with Neovascular Age-Related Macular Degeneration) aimed to evaluate the real-world effectiveness of intravitreal aflibercept injections (IVT-AFL) in Germany [8]. Patients were enrolled in 66 ophthalmological clinics and practices throughout Germany between 2013 and 2015 and followed for 24 months. In addition to the visual acuity outcomes, treatment patterns in patients with nAMD in routine clinical practice were observed.

The 12-month interim analysis of the PERSEUS study was reported in 2017 [8]. At 12 months, treatment-naïve patients obtained a mean VA gain of 5.3 letters whereas previously treated patients (who received any pretreatment for nAMD) tended to maintain their baseline VA.

However, it turned out that almost 3/4 of the total study population did not receive treatment in regular injection intervals according to the European Summary of Product Characteristics (SPC) (initial dosing of 3 monthly injections of 2 mg IVT-AFL, followed by 2 mg IVT-AFL every 2 months in year one). Remarkably, treatment pattern was associated with VA outcome at month 12: with a regular treatment scheme, treatment-naïve patients achieved an average VA gain of 8.0 letters compared to 4.0 letters with irregular treatment.

Here, we report the results of the PERSEUS study at 24 months of treatment with IVT-AFL.

## Methods

### Study design

The PERSEUS study was a prospective, non-interventional, non-controlled, multicenter, observational cohort study conducted in eye hospitals and practices throughout Germany. Patients were enrolled consecutively from July 2013 through March 2015 and were followed up for 24 months. All treatment decisions, including the decision to treat with IVT-AFL, were made by the attending physician, according to his/her experience. The study was performed in accordance with the tenets of the Declaration of Helsinki. All participants provided written informed consent.

### Eligibility

Patients with nAMD for whom treatment with IVT-AFL in accordance with the European SPC was initiated were eligible for the study.

Exclusion criteria were contraindications as listed in the European SPC (see supplementary methods). Additional exclusion criteria are as described in Framme et al. [8] and detailed in the supplementary methods.

### Objectives

The primary endpoint was the mean change in VA from baseline at 12 and 24 months. VA was assessed by the treating physician in accordance with his or her routine clinical practice; data then were converted to Snellen visual acuity ratios and Early Treatment Diabetic Retinopathy Study (ETDRS) letter score to ensure consistency as described previously (see supplementary methods, Table S1). [9, 10] Other key outcomes included the percentage of patients with improvement or worsening of ≥ 15 ETDRS letters and the number of visits, injections, and ophthalmological assessments. Data were stratified by treatment-naïve and previously treated patients. In addition, outcomes were compared between patients that were treated at regular intervals (regular cohort) and patients whose injection intervals deviated from a regular treatment regimen (irregular cohort). Regular treatment was defined as treatment in accordance with the European SPC [3]. At the time of the study, the recommended treatment scheme was as follows: initial dosing with monthly 2 mg IVT-AFL once per month for 3 months followed by bimonthly 2 mg IVT-AFL (which adds up to a total of ≥ 7 IVT-AFL at 12 months) and at least 4 injections in the second year of treatment.

To account for real-life clinical practice variability, the following windows were allowed: − 1 week/+2 weeks during initial dosing with monthly injections and − 2/+ 4 weeks with bimonthly injections, as also used in other studies of patients treated with IVT-AFL [3]. Safety data were collected as prescribed by the routine adverse event collection process.

### Statistical analyses, data sets, and missing value imputation

Statistical analyses were explorative and descriptive. The study was not aimed to confirm or reject pre-defined hypotheses.

All patients with at least one documented IVT-AFL were included in the safety analysis set (SAF). Prerequisites for inclusion in the EFF set were at least one documented IVT-AFL, a documented VA score of the study eye at baseline, and at least at one follow-up visit. Details on treatment time frames are provided in the supplementary methods. To account for missing data, the primary endpoint (mean VA change from baseline) was analyzed using both the last observation carried forward (LOCF) approach and the observed cases (OC) method. For the LOCF method, the last measurement within ± 30 days (± 15 days during upload) around the time points of analysis was included in the analysis. If no measurements have been taken at that time, the last measurement recorded was taken into account if it occurred 120 days or later after the first injection. In the OC method, only patients with an available VA assessment at 24 months were considered in the analysis. In addition, the composition of the regular and irregular treatment cohorts was either fixed at 12 months to evaluate changes of the 12-month study population over the second treatment year or free up to month 24. Thus, four populations were analyzed:

1. Fixed LOCF population: The LOCF approach was used to impute missing VA data. The composition of the regular and irregular cohorts was fixed at month 12. This approach allows evaluation of changes in the actual 12-month study populations over 24 months. Thus, patients that were irregularly treated in the second year remained in the regular cohort.
2. Free LOCF population: The LOCF approach was used to account for missing VA data. The regular cohort included only patients that received regular treatment for the entire follow-up period of 24 months. Patients that were irregularly treated in the second year were assigned to the irregular cohort.
3. Fixed OC population: Only patients with available VA at 24 months were considered. The composition of the regular and irregular cohorts was fixed at month 12. This approach allows evaluation of changes in the actual 12-month study populations over 24 months. Thus, patients that were irregularly treated in the second year remained in the regular cohort.
4. Free OC population: Only patients with available VA at 24 months were considered. Patients that were irregularly treated in the second year were assigned to the irregular cohort.

The Wilcoxon rank-sum test was used to compare changes in VA between patient groups. To investigate the association between selected covariates and change in VA letter score between baseline and follow-up after 24 months, linear regression was performed. First, univariate linear regression was performed for the dependent variable change in VA letter score after 24 months. Afterward, all independent covariates (baseline VA letter score, age at indication, gender, and baseline central retinal thickness [CRT], baseline lesion characteristics measured by fluorescein angiography (FA), and pretreatment and regular or irregular treatment defined by deviations from the treatment scheme as specified in the SPC) were entered into a stepwise multivariate linear regression. The entry level was *P* = 0.5 and the stay level was *P* = 0.05. All remaining significant covariates were considered to be associated with the change in VA letter score after 24 months. To avoid different sample sizes, missing observations were dropped for univariate and multivariate regression, respectively.

In further analyses, logistic regression was used to investigate the association between baseline covariates and regular or irregular treatment. To determine the association, univariate logistic regression was performed for the dependent variable regular or irregular treatment with the outcomes of regular and irregular. Afterward, all independent covariates (baseline VA letter score, age at indication, gender, baseline CRT, lesion type on FA, and previous treatment) were entered into a stepwise multivariate logistic regression for the above-mentioned dependent variable. The entry level was *P* = 0.5 and the stay level was *P* = 0.05.

Safety was assessed on the safety set, which included all patients who received at least 1 IAI treatment.

## Results

### Patient disposition and baseline characteristics

**Fig. 1:**
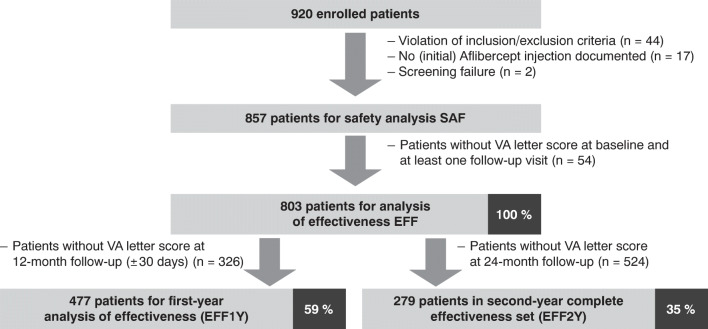
Patient disposition and data sets. EFF analysis of effectiveness, EFF1Y first-year complete effectiveness set, EFF2Y second-year complete effectiveness set, SAF safety analysis set, VA visual acuity. Adobe Illustrator CC 2020 was used to create the artwork

The PERSEUS study enrolled a total of 920 patients in 66 study sites throughout Germany. Figure 1 depicts patient disposition. 803 patients were included in the analysis of effectiveness (EFF). The composition of the 24-month EFF set differs from the EFF set in the 12-month interim analysis [8]. At month 24, 279 patients had a documented VA test (Fig. 1). Of those, 149 (53.4%) had been treatment-naïve while 130 (46.6%) had previously received treatment for nAMD. During the 24-month treatment period, 578 patients of the SAF discontinued the study (see supplementary results).

Table 1 summarizes the baseline characteristics of the 24 months EFF set (*n* = 803). 44.2% of the EFF set had received previous treatment with a mean duration of 15.5 (SD = 17.8) months (ranibizumab only: 69.9%, off-label bevacizumab only: 19.7%, ranibizumab and bevacizumab 6.5%; other: 4.2%).

**Table 1:** Baseline characteristics of the effectiveness set (EFF), *N* = 803

	Treatment-naïve (*n* = 448)	Previously treated (*n* = 355)	Total (*n* = 803)
Age, mean (SD)	77.5 (7.7)	77.8 (7.9)	77.6 (7.8)
Female, *n* (%)	279 (62.3)	219 (61.7)	498 (62.0)
CNV type, *n* (%):			
Predominantly classic	147 (32.8)	58 (16.3)	205 (25.5)
Minimally classic	28 (6.25)	16 (4.5)	44 (5.5)
Occult, with no classic	133 (29.7)	64 (18.0)	197 (24.5)
No CNV or CNV not active	1 (0.2)	2 (0.6)	3 (0.4)
CNV type unknown	18 (4.0)	2 (0.6)	20 (2.5)
No baseline FA available	121 (27.0)	213 (60.0)	334 (41.6)
Central retinal thickness (mean)	359.2 μm	341.3 μm	351.1 μm
ETDRS letter score, mean (SD) US Snellen score	52.9 (18.1) 20/80	53.1 (18.2) 20/80	53.0 (18.1) 20/80

*CNV* choroidal neovascularization, *ETDRS* Early Treatment Diabetic Retinopathy Study, *FA* fluorescein angiography, *SD* standard deviation

### Treatment patterns and injection numbers

**Fig. 2:**
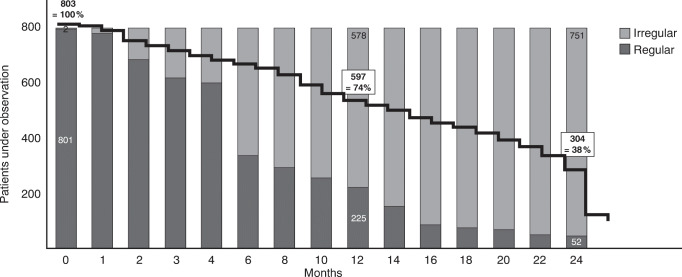
Shift from regular to irregular treatment over the course of 24 months (total EFF set). Patients who discontinued the study or were lost to follow-up are included in the irregular group. The black line represents the number of patients under observation at each time point. EF F analysis of the effectiveness. Adobe Illustrator CC 2020 was used to create the artwork

Over the course of 24 months, there was a shift from regular to irregular treatment in the EFF set. While the regularly treated cohort predominated up to month 4, from month 6 on, most patients were either treated irregularly or had discontinued (Fig. 2). While 74% (*n* = 597) of the patients were still under observation at month 12, persistence had dropped to 38% (*n* = 304) at month 24.

Within 24 months, treatment-naïve and previously treated patients received a similar mean number of injections (treatment-naïve [*n* = 448]: 8.0 [± 3.8] and previously treated [*n* = 355]: 8.1 [± 4.3] injections). Regularly treated patients received a higher number of injections than irregularly treated patients (regular [*n* = 52]: 13.1 (± 1.3), irregular [*n* = 751]: 7.8 [± 4.09]).

### Mean change in visual acuity

At 24 months, the mean VA change from baseline was analyzed using two approaches for handling of missing data: the LOCF method and the observed cases (OC) method. Additionally, the composition of the regular and irregular treatment cohorts was either fixed at 12 months or free up to month 24 (see Methods for a detailed description).

**Fig. 3:**
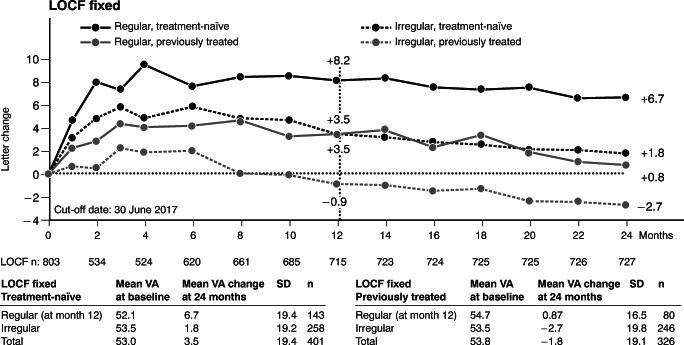
VA change from baseline over 24 months in the fixed LOCF population. Composition of regular and irregular cohorts was fixed at month 12 to evaluate changes of the 12-month group over 24 months. LOCF last observation carried forward, SD standard deviation, VA visual acuity. Adobe Illustrator CC 2020 was used to create the artwork

The LOCF population comprised 727 patients. The analysis of the fixed LOCF population allows evaluation of changes in the actual 12-month study populations over 24 months (Fig. 3). Among treatment-naïve patients, the fixed regular cohort (*n* = 143) achieved a mean VA increase of 6.7 letters at month 24 compared to 1.8 letters in the fixed irregular cohort (*n* = 258). Among previously treated patients, the fixed regular cohort (*n* = 80) showed a mean VA change of 0.8 letters at month 24 versus − 2.7 letters in the fixed irregular cohort (*n* = 246). The mean VA gain of the total LOCF population was + 1.1 letters (treatment-naïve: + 4.3 letters, previously treated: − 1.0 letters).

Overall, the VA trends observed during the second treatment year remained comparable to those observed in the first year (Fig. 3). For example, treatment-naïve patients with regular treatment obtained the best VA outcomes and could virtually maintain their VA over the second year. Previously treated patients in the irregular cohort experienced a decrease in VA from month 6 on which continued during the second treatment year (Fig. 3).

Table 2 summarizes the VA outcomes at 24 months obtained in the fixed and free LOCF and OC populations. Remarkably, treatment-naïve patients in the OC population that received regular treatment achieved mean VA changes of 8.2 (fixed regular cohort, *n* = 51) and 8.1 (free regular cohort, *n* = 18) letters (Table 2).

**Table 2:** Mean VA change from baseline in LOCF and OC populations at month 24

	Mean VA change from baseline at month 24			
	LOCF population (*n* = 727)		OC population (*n* = 279)	
	Fixed	Free	Fixed	Free
Treatment-naïve				
Regular	6.7 (*n* = 143)	6.3 (*n* = 27)	8.2 (*n* = 51)	8.1 (*n* = 18)
Irregular	1.8 (*n* = 258)	3.3 (*n* = 374)	1.9 (*n* = 98)	3.6 (*n* = 131)
Previously treated				
Regular	0.8 (*n* = 80)	5.8 (*n* = 25)	1.4 (*n* = 40)	4.2 (*n* = 18)
Irregular	− 2.7 (*n* = 246)	− 2.4 (*n* = 301)	− 2.3 (*n* = 90)	− 2.1 (*n* = 112)

*LOCF* last observation carried forward, *OC* observed cases, *VA* visual acuity

### Gain and loss of ≥ 15 letters

**Fig. 4:**
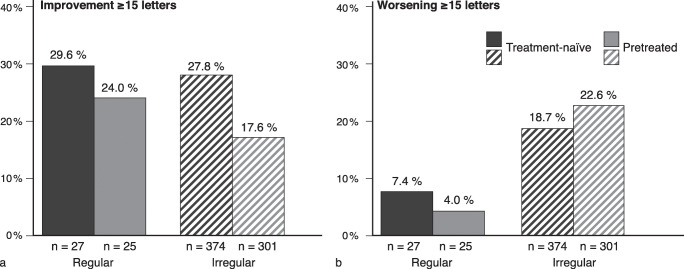
Proportion of patients with **a** improvement ≥ 15 ETDRS letters or **b** worsening of ≥ 15 ETDRS letters (LOCF population, *N* = 727). Numbers represent the patient population in the respective cohort. ETDRS Early Treatment Diabetic Retinopathy Study, LOCF last observation carried forward. Adobe Illustrator CC 2020 was used to create the artwork

The proportion of patients with a gain or loss of 15 letters or more was evaluated in the LOCF population (*n* = 727). The proportion of treatment-naïve patients achieving a VA improvement of ≥ 15 letters did not differ profoundly between regular and irregular treatment (Fig. 4a). However, the proportion of patients with a VA worsening of ≥ 15 letters was considerably higher in the irregular cohort compared to the regular cohort (Fig. 4b). Among treatment-naïve patients, 18.7% experienced a loss of ≥ 15 letters with irregular treatment, compared to 7.4% with regular treatment (Fig. 4b).

### Other secondary outcomes

The average number of visits, post-injection visits, VA tests, and OCT measurements was consistently higher in the first year compared to the second year (visits: 9.3 vs. 4.8; post-injection visits 4.8 vs. 1.9; VA tests: 11.2 vs. 5.4; OCTs: 4.3 vs. 2.6).

**Fig. 5:**
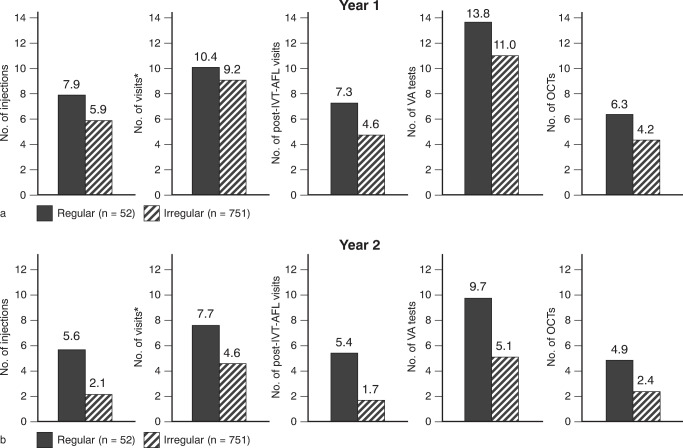
Number of injections, visits*, post-IVT-AFL visits, VA tests, and OCTs within **a** the first and **b** the second year of IVT-AFL treatment in regularly and irregularly treated patients (EFF set, *N* = 803). *Visits include injection visits, control visits, and combined visits. EFF analysis of effectiveness, IVT-AFL intravitreal aflibercept injections, OCT optical coherence tomography, VA visual acuity. Adobe Illustrator CC 2020 was used to create the artwork

Within both treatment years, the mean numbers of injections, of visits (including visits for injections, monitoring, and a combination of both), of post-IVT-AFL visits (only for safety checks after injection), of VA tests, and of OCT tests was considerably lower in the irregular cohort compared to the regular cohort (Fig. 5). Outcomes from CRT measurements are provided in the supplementary results.

### Safety analysis

The observed safety profile of IVT-AFL was in line with previously obtained safety data (see supplementary results).

## Discussion

The results of the PERSEUS study at 24 months demonstrate that the outcomes obtained after the first treatment year [8] can be largely maintained in a subset of treatment-naïve patients that receive regular treatment over a 2-year period in routine clinical practice. However, this was true only for a minority of patients, as the study revealed that in clinical practice in Germany, irregular treatment and discontinuation of treatment are common. At 24 months, only 38% of the patients were still under observation. Of those, more than 80% had received irregular treatment during the follow-up period. Irregular treatment was associated with lower VA gains and with a higher risk of worsening of 15 letters or more at 24 months compared to patients that received regular treatment.

Limitations of the PERSEUS study arise from its observational, non-controlled design. There was a wider range of baseline VA and a greater range of other health issues than in the phase-3-studies for IVT-AFL [3]. Furthermore, patients who had previously received other nAMD treatment were included. Since there was no fixed visiting schedule, the timing of measurements varied between patients, and the extent of collected data depended on routine practice in the study centers. The high study discontinuation rate of up to 62% at month 24 is a potential source of bias. The strengths of the PERSEUS study include its large number of enrolled participants and its prospective multicenter design. Two different analytical methods (OC and LOCF) were used for handling missing data to provide a more objective estimation of the treatment effect in primary endpoint analysis. Since patients were enrolled in different types of healthcare facilities, PERSEUS represents the medical care situation reasonably well.

As expected, the analysis of the VA change from baseline by the LOCF and OC methods yielded slightly different results. However, the main features observed for the different patient populations were similar. First, previously treated patients benefited less from IVT-AFL treatment than treatment-naïve patients, probably because in previously treated patients, the disease is generally more advanced. Second, regularly treated patients obtained better outcomes than irregularly treated patients.

In the treatment-naïve regular cohorts, the OC population obtained better results than the LOCF population (e.g., 8.2 vs. 6.7 letters in the fixed cohorts). The VA in the regularly treated OC population was similar to the VA gains obtained under clinical trial conditions in the VIEW studies [3]. Although this is a small and selected population, this indicates that it is possible to obtain optimal outcomes can also under real-world conditions.

In general, the LOCF approach often leads to an underestimation of the treatment effect, since it is assumed that a patient’s response would have been constant from the last observed value to the end of the follow-up period and it is more likely that patients with an unfavorable response drop out [11]. In the OC approach, on the other hand, the overall treatment effect may be overestimated, since only patients that respond well to the treatment complete the study. Both the LOCF and OC population reflect certain aspects of the real-life situation and we may assume that both contribute to the “true” overall treatment effect. The OC population demonstrates that in patients that receive regular treatment over 2 years, optimal VA outcomes can be achieved. The LOCF population on the other hand also comprises patients who discontinued the study during the 2-year treatment period. Among those, there may be patients who felt that the effect of their treatment did not meet their expectations and thus they became non-adherent, leading to worsening results, or vice versa, patients that did not achieve optimal results due to insufficient adherence. The importance of a consistent and regular treatment approach to obtain the best possible VA outcomes is further underlined by a recently published post hoc analysis of the PERSEUS 12-month data that included only patients that had received at least the number of injections required in a regular treatment scheme (≥ 7 injections during the first treatment year). 43.5% of the patients that had received ≥ 7 injections had experienced irregular treatment, which was associated with inferior VA results compared to the regularly treated group [12].

When interpreting the results for patients with gain or loss of ≥ 15 letters in the LOCF population at 24 months, it must be taken into account that in most of the previous studies on anti-VEGF treatment for nAMD, the VA decreases in the second year [1–3]. Thus, the proportion of patients gaining ≥ 15 letters may be overestimated whereas the proportion of patients with a loss of ≥ 15 letters may be underestimated.

Compared to the AURA study, which describes a real-world experience with ranibizumab treatment in treatment-naïve patients in Germany [5], PERSEUS provides evidence for an improvement of care in the treatment of patients with nAMD. In the PERSEUS study, better functional results were observed at the end of the second treatment year (treatment-naïve (LOCF): + 4.3 letters versus AURA 0.8 letters) [5]. Furthermore, patients received a higher number of intravitreal injections (8.1 ± 4.1 versus 5.6 ± 3.5) and visits were carried out more frequently (PERSEUS year 1: 9.3 and year 2: 4.8; AURA year 1: 7.8; year 2: 3.1) [5].

Despite this virtual improvement, the outcomes of the PERSEUS study still underline the major obstacles in clinical practice in nAMD treatment: a high percentage discontinues treatment within the first two treatment years despite the chronic nature of the disease. Furthermore, the majority of patients remaining on treatment do not receive injections in a sufficient frequency to ensure optimal results.

Similar to the PERSEUS study, other German real-world studies on anti-VEGF treatment other than aflibercept describe high rates of both non-persistence and non-adherence [13–15]. In the OCEAN study, only 29% of nAMD patients receiving ranibizumab were still on therapy at 24 months of treatment [15]. In the PONS study, which analyzed adherence and persistence to pegaptanib, bevacizumab and ranibizumab for treatment of nAMD, 95.6% of the patients fulfilled the criteria for non-adherence (defined as no treatment or consultation with a VA measurement and OCT at least every 6 weeks) after 12 months of treatment. 18.2% were non-persistent (e.g., had no ophthalmological consultation for more than 3 months) [14]. Another study on real-life VA outcomes in patients that receive anti-VEGF treatment for nAMD, diabetic macular edema, or branch retinal vein occlusion in Germany identified patient-associated non-adherence to treatment and follow-up regimens as a major factor limiting clinical treatment outcomes under real-world conditions [13]. Thus, non-adherence and non-persistence seem to be common in VEGF treatment irrespective of the indications and the substance used.

Although the reasons for irregular treatment in the PERSEUS study were not evaluated, we can speculate that insufficient treatment adherence plays a major role. Furthermore, we can assume that irregular treatment could also be due to the fact that for most of the study period pro-re-nata (PRN) treatment schemes were actively endorsed by the German professional associations [16], as has already been discussed in the PERSEUS 12-month publication [8]. In PERSEUS, the rate of patients with irregular treatment increases sharply following the upload phase (with a slight delay due to the permitted time windows). Similarly, non-adherence in the PONS study doubles in months 3 to 6 [14]. It seems thus important that both patients and caregivers are aware of the chronicity of the disease and that continued treatment is required to maintain the VA improvement achieved in the initial treatment phase.

The high rate of non-adherence and non-persistence observed in real-world anti-VEGF treatment is particularly worrying, since nAMD is commonly referred to as an “unforgiving disease” and loss of visual acuity is often irreversible. Most patients are in fact expected to require lifelong therapy. Thus, it appears crucial that patient management in nAMD treatment focuses more on maintaining the patient’s adherence and persistence. [17]

In June 2018, the European Aflibercept SPC was updated, allowing for a treat-and-extend approach during the first year of treatment. The controlled randomized ALTAIR study evaluated the efficacy and safety of IVT-AFL in T&E regimens in 288 Japanese patients. Patients received three initial monthly doses of IVT-AFL 2 mg in the study eye. Patients received IVT-AFL at week 16 and were randomized 1:1 to receive IVT-AFL in a T&E regimen with either a 2-week or 4-week adjustment. At 52 weeks, both regimens had improved functional and anatomic outcomes (VA + 9.0 and + 8.4 letters; CRT − 134.4 and – 126.1 μm) and up to 40% of the patients had reached the intended injection interval of 16 weeks [19].

It will be interesting to see if T&E regimens with their reduced treatment burden compared to a fixed administration regimen can positively influence adherence and persistence to IVT-AFL treatment in a real-world setting. To this end, the non-interventional study ANDROMEDA, which started in January 2019, aims to gain a deeper understanding of treatment consistency and to identify potential reasons for non-adherence and non-persistence in patients with nAMD [18].

## Supplementary information

ESM 1 (PDF 21 kb)

ESM 2 (PDF 79 kb)

## Data Availability

Not applicable.

## Abbreviations

ALTAIR: Japanese treat and extend study of aflibercept in neovascular age-related macular degeneration
ANDROMEDA: intravitreal aflibercept in neovascular age-related macular degeneration: an observational study assessing patient relevant outcomes, real-world treatment pattern and effectiveness
AURA: a retrospective non-interventional study to assess the effectiveness of existing anti-vascular endothelial growth factor treatment regimens in patients with wet age-related macular degeneration
COMPASS: study to assess the efficacy of treatment with ranibizumab in patients with wet age-related macular degeneration in routine clinical care
IVT-AFL: intravitreal aflibercept
nAMD: neovascular age-related macular degeneration
PERSEUS: a prospective non-interventional study to assess the effectiveness of intravitreal aflibercept in routine clinical practice in Germany in patients with neovascular age-related macular degeneration
PRN: pro-re-nata
T&E: treat-and-extend
VA: visual acuity
VEGF: vascular endothelial growth factor
VIEW: VEGF Trap-Eye: investigation of efficacy and safety in wet age-related macular degeneration
WAVE: Lucentis in wet age-related macular degeneration: evaluation of visual acuity and quality of life
