# Supplementary material for: PERSEUS 24-month analysis: a prospective non-interventional study to assess the effectiveness of intravitreal aflibercept in routine clinical practice in Germany in patients with neovascular age-related macular degeneration
Source: Graefes Arch Clin Exp Ophthalmol. 2021 Feb 6;259(8):2213–23. doi: 10.1007/s00417-021-05073-8 (PMC8352822; doi:10.1007/s00417-021-05073-8)
Supplement: Supplementary file 1 — (PDF 21 kb) [file 417_2021_5073_MOESM1_ESM.pdf]

PERSEUS 24-Month analysis: A Prospective Non-interventional Study to Assess the Effectiveness of Intravitreal Aflibercept in Routine Clinical Practice in Germany in Patients with Neovascular Age-related Macular Degeneration

Graefe's Archive for Clinical and Experimental Ophthalmology

Nicole Eter, MD, Zoran Hasanbasic, MD, Georgios Keramas, PhD, Christine Rech, PhD, Helmut Sachs, MD, Harald Schilling, MD, Joachim Wachtlin, MD, Peter Wiedemann, MD, Carsten Framme, MD for the PERSEUS Study Group.

Corresponding author:

Univ.-Prof. Dr. med. Nicole Eter

Universitäts-Augenklinik Münster

Domagkstr. 15, 48149 Münster, Germany

Tel: +49 – 251 – 83 56004, Fax: +49 – 251 – 83 56003, Email: eter@uni-muenster.de

Supplementary methods:

Eligibility

nAMD patients for whom treatment with IVT-AFL in accordance with the local SPC was initiated were eligible for the study. Exclusion criteria were as listed in the local SPC: hypersensitivity to the active substance aflibercept or to any of the excipients, active or suspected ocular or periocular infection, and active severe intraocular inflammation. In addition, patients with scarring, fibrosis, or atrophy involving the center of the fovea or who were treated for nAMD with any other agent in the study eye were excluded. Eyes with retinal pigment epithelium tears, detachment, or lesion of the retinal pigment epithelium were eligible. Previous treatment for nAMD, including treatment with anti-VEGF agents (ranibizumab, bevacizumab, pegaptanib), was permitted. A washout period (previously treated patients) before initiation of IVT-AFL treatment was not required.

Statistics – allowed time windows

Because of the observational nature of this study, there was no fixed visit schedule. Consequently, the timing of measurements varied more between patients than it would have in a randomized clinical trial, and the extent of collected data depended on clinical practice in the study centers. During treatment initiation (until month 2), a predefined window of ±15 days was allowed for all time points; for the subsequent maintenance phase, this window was broadened to ±30 days. Therefore, the number of included patients may vary for different end points. Changes from baseline in VA and central retinal thickness were analyzed for time points equivalent to months 1 and 2, as well as month 4 and were recorded every two months up until month 24.

*Table S1: conversion of Decimal, US Snellen and logMAR units into Visual acuity letter score**

Visual acuity score (letter count)	Decimal	US SNELLEN	logMar
95	1.6	20/12.5	-0.2
90	1.25	20/16	-0.1
85	1.0	20/20	0.0
80	0.8	20/25	0.1
75	0.63	20/32	0.2
70	0.5	20/40	0.3
65	0.4	20/50	0.4
60	0.32	20/63	0.5
55	0.25	20/80	0.6
50	0.2	20/100	0.7
45	0.16	20/125	0.8
40	0.125	20/160	0.9
35	0.10	20/200	+1.0
30	0.08	20/250	1.1
25	0.063	20/320	1.2
20	0.05	20/400	1.3
15	0.04	20/500	1.4
10	0.03	20/630	1.5
5	0.025	20/800	1.6
No letter count; hand	0.02	20/1000	1.7
motion only	0.016	20/1250	1.8
	0.0125	20/1600	1.9
	0.01	20/2000	2.0

logMAR = logarithm of the minimum angle of resolution

* Gregori NZ, Feuer W, Rosenfeld PJ. Novel method for analyzing snellen visual acuity measurements. Retina 2010;30(7):1046-50.
