# Supplementary material for: PERSEUS 24-month analysis: a prospective non-interventional study to assess the effectiveness of intravitreal aflibercept in routine clinical practice in Germany in patients with neovascular age-related macular degeneration
Source: Graefes Arch Clin Exp Ophthalmol. 2021 Feb 6;259(8):2213–23. doi: 10.1007/s00417-021-05073-8 (PMC8352822; doi:10.1007/s00417-021-05073-8)
Supplement: Supplementary file 2 — (PDF 79 kb) [file 417_2021_5073_MOESM2_ESM.pdf]

PERSEUS 24-Month analysis: A Prospective Non-interventional Study to Assess the Effectiveness of Intravitreal Aflibercept in Routine Clinical Practice in Germany in Patients with Neovascular Age-related Macular Degeneration

Graefe's Archive for Clinical and Experimental Ophthalmology

Nicole Eter, MD, Zoran Hasanbasic, MD, Georgios Keramas, PhD, Christine Rech, PhD, Helmut Sachs, MD, Harald Schilling, MD, Joachim Wachtlin, MD, Peter Wiedemann, MD, Carsten Framme, MD for the PERSEUS Study Group.

Corresponding author:

Univ.-Prof. Dr. med. Nicole Eter

Universitäts-Augenklinik Münster

Domagkstr. 15, 48149 Münster, Germany

Tel: +49 – 251 – 83 56004, Fax: +49 – 251 – 83 56003, Email: eter@uni-muenster.de

Supplementary results:

Reasons for study discontinuation

578 patients of the SAF discontinued the study. The main reasons for discontinuing the study were permanent discontinuation of treatment (14.8%), switch of treatment (14.8%), loss to follow-up (14.5%) and change of treating physician (4.7%). Adverse events were stated in only 1.4% as a reason for discontinuation.

Anatomic outcomes

Data on central retinal thickness (CRT) was available for 232 patients of the treatment-naïve cohort and 203 patients of the previously treated cohort. At baseline, the treatment-naïve cohort had a mean CRT of 359.2 µm which at 12 months was reduced by -90.1 µm. This reduction in CRT was nearly maintained until 24 months (-86.7±108.6 µm compared to baseline). The previously treated cohort had a mean CRT of 341.3 µm at baseline which was reduced by -59.0 µm at month 12 and could be further decreased to -62.3±130.2 µm.

Safety

The safety set (SAF) included data from 857 patients. A total number of 7,390 injections was administered during the 24-month study period. 4.4% of all patients experienced non-ocular treatment-emergent adverse events (TEAE). 10.7% experienced ocular TEAEs including cataract (3.3% of all patients), conjunctival hemorrhage (1.3% of all patients) and corneal erosion (0.8% of all patients). No cases of endophthalmitis occurred. The table details the documented TEAEs.

Table 1: Safety analysis (n = 857)

**Event***		**N**	**%**
TEAE		118	13.8
Non-ocular TEAE		38	4.4
Ocular TEAE		92	10.7
	Cataract	28	3.3
	Conjunctival hemorrhage	11	1.3
	Corneal erosion	7	0.8
Drug-related ocular TEAE		31	3.6
	Conjunctival hemorrhage	10	1.2
	Lacrimation increased	3	0.4
	Anterior chamber cell	3	0.4
Serious ocular TEAE		9	1.1
	Cataract	2	0.2
	Retinal pigment epithelial tear	2	0.2
	Macular fibrosis	2	0.2
Retinal epithelial tear		4	0.5
Arterial thromboembolic event**		4	0.5
Endophtalmitis		0	0.0

TEAE = treatment-emergent adverse event (adverse events that occurred after first IVT-AFL and until 30 days after last IVT-AFL injection).

* The three most common documented events; **transient ischaemic attack in one patient with a medical history of a prior arterial thromboembolic event
